# Rehabilitative and Preventive Effects of the Thrower’s Ten Program in Overhead Athletes: A Systematic Review

**DOI:** 10.7759/cureus.95081

**Published:** 2025-10-21

**Authors:** Purvi Patel, Jayesh Vaishnav

**Affiliations:** 1 Musculoskeletal and Sports Physiotherapy, College of Physiotherapy, Sumandeep Vidyapeeth Deemed to be University, Vadodara, IND; 2 Sports Physiotherapy, College of Physiotherapy, Sumandeep Vidyapeeth Deemed to be University, Vadodara, IND

**Keywords:** injury prevention, overhead athletes, racket sports, rotator cuff strength, scapular stabilization, shoulder rehabilitation, sports performance, thrower’s ten

## Abstract

Overhead athletes are repeatedly exposed to high-velocity arm motions that impose significant stress on the shoulder complex, predisposing them to dysfunction and injury. The Thrower’s Ten (T10) program was originally developed as a rehabilitation protocol but has since been adopted for injury prevention and performance enhancement in overhead sports. This systematic review synthesized evidence from eight studies published between 2015 and 2025 that evaluated the effects of T10 or its modified versions in cricket, handball, baseball, badminton, shot put, and mixed overhead athletes. The included designs comprised randomized controlled trials, controlled trials, quasi-experimental studies, pre-post interventions, and a feasibility pilot. Across these diverse contexts, the program was consistently associated with improvements in scapular stability, shoulder range of motion, rotator cuff strength, endurance, and sport-specific performance. Evidence also supports the feasibility of implementing T10 in youth and team training settings. Collectively, the findings position the Thrower’s Ten as a versatile, low-cost, and evidence-based intervention that extends beyond rehabilitation to encompass injury prevention and performance enhancement. The program’s adaptability, minimal equipment requirements, and demonstrated efficacy make it highly suitable for athletes across multiple overhead sports.

## Introduction and background

Overhead athletes represent a unique population within sports medicine because their activities demand repetitive high-velocity arm motions that impose extreme stresses on the glenohumeral joint. Sports, such as baseball, tennis, badminton, volleyball, handball, cricket, and swimming, involve thousands of overhead actions during training and competition. These movements place high eccentric and concentric loads on the rotator cuff and scapular stabilizers, and over time, can lead to pain, dysfunction, and injury. The shoulder, designed for mobility rather than bony stability, relies heavily on dynamic stabilizers to maintain function, which increases vulnerability to overuse injuries [1-4].

Epidemiological studies confirm the prevalence of shoulder pathology in overhead athletes. In tennis, nearly one-third of musculoskeletal complaints involve the shoulder, and serve mechanics produce very high angular velocities [5]. In badminton, smash velocities can exceed 300 km/h, producing repetitive eccentric strain on the rotator cuff and posterior capsule [6]. Volleyball players, particularly spikers and servers, demonstrate substantial rates of shoulder injury and time lost from competition [7]. Similarly, cricket fast bowlers frequently present glenohumeral internal rotation deficits (GIRD) and posterior shoulder tightness comparable with baseball pitchers [8]. Handball athletes sustain cumulative shoulder loading from frequent throwing combined with contact, while swimmers execute thousands of overhead strokes that predispose them to multi-directional instability and high rates of shoulder pain [9,10]. Collectively, these sports highlight the cumulative mechanical burden on the shoulder complex.

The biomechanics of throwing explain the mechanisms underlying injury risk. The sequence of wind-up, cocking, acceleration, deceleration, and follow-through exposes the glenohumeral joint to extreme ranges and forces. During late cocking the shoulder externally rotates markedly, while acceleration and deceleration phases involve concentric and eccentric contractions at very high angular velocities and substantial eccentric loads on the posterior cuff and scapular stabilizers. Insufficient strength, endurance, or neuromuscular coordination in these muscles predisposes athletes to labral pathology, rotator cuff overload, impingement syndromes, and instability [11-13].

Earlier rehabilitation programs concentrated on isolated rotator cuff strengthening but often neglected scapular mechanics and kinetic chain contributions. A conceptual shift toward integrated approaches (scapular control, trunk/core integration, neuromuscular coordination) emerged in the early 2000s and has been championed by authors such as Kibler and Cools. Restoring scapular muscle balance and neuromuscular control is now recognized as essential for both injury prevention and performance optimization [3,14].

Within this context, Wilk et al. developed the Thrower’s Ten (T10) exercise series in the early 1990s, followed by later adaptations, such as the Advanced Thrower’s Ten and the Youth Thrower’s Ten. These programs target the rotator cuff, scapular stabilizers, and forearm musculature, requiring minimal equipment and making them accessible across all levels of sport [15-17].

Despite broad clinical adoption, rigorous evaluation of T10 in athlete samples was limited until the last decade. More recently, indexed intervention studies have assessed their rehabilitative, preventive, and performance effects across overhead sports; however, the evidence is heterogeneous. This systematic review, therefore, aimed to identify and critically appraise trials of Thrower’s Ten (and clearly described modifications) in overhead athletes published between 2015 and 2025, focusing on outcomes including strength, range of motion (ROM), scapular stability, functional performance, and injury prevention.

## Review

Methodology

This systematic review followed the Preferred Reporting Items for Systematic Reviews and Meta-Analyses (PRISMA) 2020 guidelines [18]. This systematic review was registered prospectively in the International Prospective Register of Systematic Reviews (PROSPERO), registration number #CRD420251143009. The objective was to synthesize empirical evidence on the effects of the Thrower’s Ten program in overhead athletes.

Search strategy

Electronic databases (PubMed, Scopus, Web of Science, Physiotherapy Evidence Database (PEDro), and Google Scholar) were searched from January 2015 to March 2025. The following keywords and Boolean operators were used: (“Thrower’s Ten” OR “Throwers Ten” OR “Advanced Thrower’s Ten” OR “Youth Thrower’s Ten”) AND (“overhead athlete*” OR tennis OR badminton OR volleyball OR cricket OR handball OR swimming OR “water polo” OR “shot put” OR baseball). Reference lists of included articles and relevant reviews were also screened manually to identify additional eligible studies.

The inclusion criteria were clearly defined. Eligible participants were athletes engaged in overhead or overhead-like sports. Interventions consisted of the Thrower’s Ten program or its modified versions, including advanced or youth adaptations, as well as studies where Thrower’s Ten was a core component of a combined protocol. Eligible outcomes included strength, range of motion, scapular stability, pain, functional performance, injury prevention, or feasibility of implementation. Study designs considered for inclusion were randomized controlled trials, controlled trials, quasi-experimental studies, prospective cohorts, pre-post intervention case series, or structured feasibility studies. Only articles published between 2015 and 2025 were considered, and studies were required to be available in full-text English or provide sufficient methodological details in English abstracts. Studies were excluded if they were reviews, editorials, or expert opinions rather than original research. Articles conducted in non-athlete or sedentary populations were not considered. Protocols focusing exclusively on surgical rehabilitation without a stand-alone Thrower’s Ten component were excluded. Case reports with fewer than five participants were also excluded from the review.

Study selection

**Figure 1:**
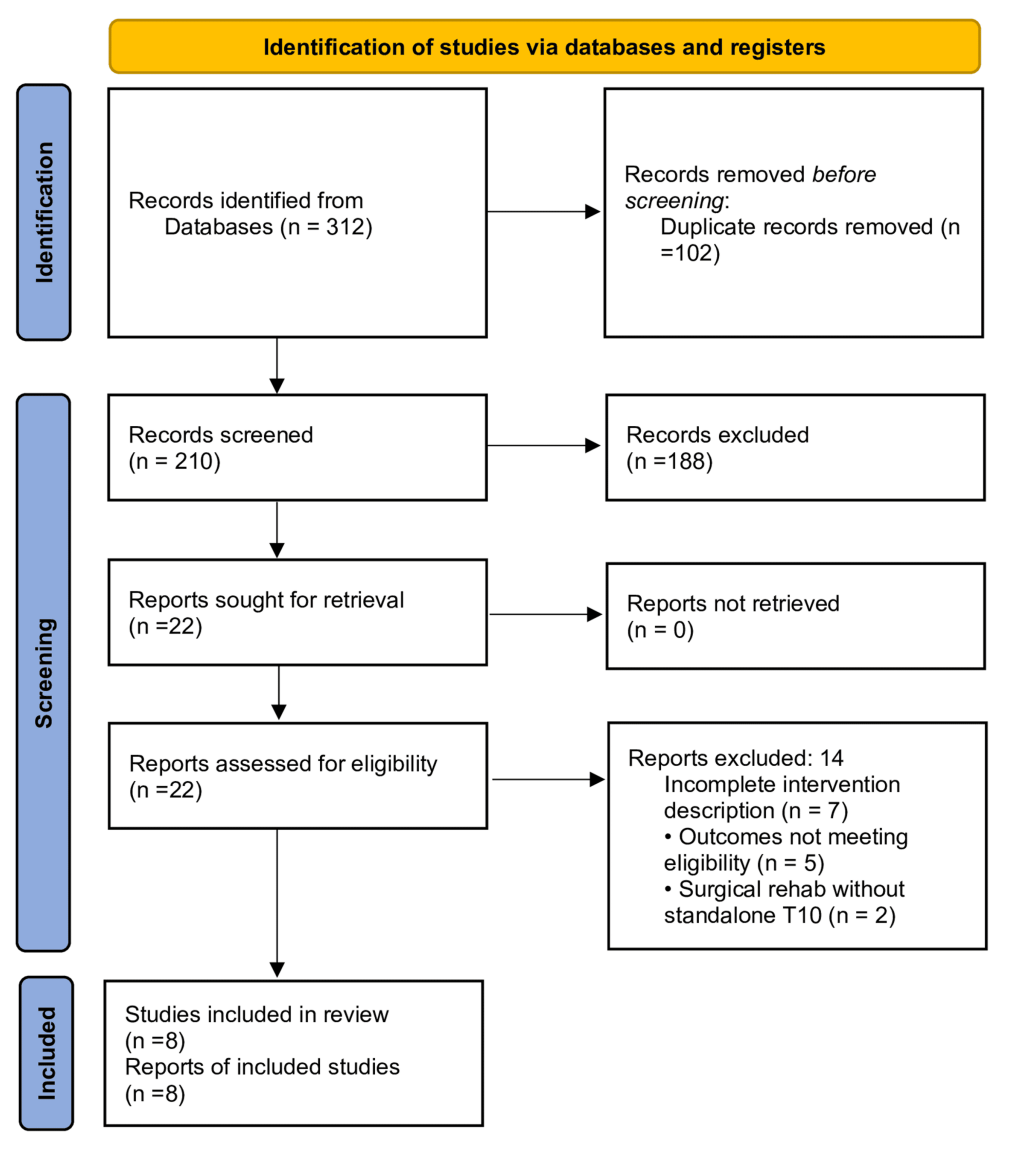
PRISMA flowchart describing search strategy. PRISMA: Preferred Reporting Items for Systematic Reviews and Meta-Analyses

A total of 312 records were identified. After removal of 102 duplicates, 210 titles/abstracts were screened. Of these, 188 were excluded (reviews, non-athlete populations, irrelevant interventions, or outside publication period). Twenty-two full-texts were assessed for eligibility. Fourteen were excluded (incomplete intervention description, outcomes not meeting eligibility criteria, or surgical rehabilitation without a stand-alone T10). Finally, eight studies were included in the synthesis [19-26]. The selection process is depicted in the PRISMA flow diagram (Figure 1).

Data extraction

Two reviewers independently extracted information on author, year, study design, sport and sample size, intervention protocol, outcomes, and key findings. Any disagreements were resolved by consensus. Methodological quality was assessed using study-specific tools. Randomized controlled trials were evaluated with the Physiotherapy Evidence Database (PEDro) scale [27]. Non-randomized controlled and quasi-experimental studies were assessed using the Newcastle-Ottawa Scale (NOS) [28]. Pre-post case series and feasibility studies were appraised using the Joanna Briggs Institute (JBI) critical appraisal checklists [29].

Data synthesis

Due to heterogeneity in sports, intervention variants, and outcomes, a narrative synthesis was performed. Results were grouped into rehabilitative, preventive, and performance-related outcomes.

Results

Study Selection

A total of eight studies met the eligibility criteria and were included in the synthesis (Table 1) [19-26]. The screening process is detailed in the PRISMA flow diagram (Figure 1).

**Table 1:** Summary of the published studies (2015-2025) evaluating the effects of the Thrower’s Ten program in overhead athletes. LSST: lateral scapular slide test; FTPI: functional throwing performance index; ROM: range of motion; CKCUEST: Closed Kinetic Chain Upper Extremity Stability Test; SMBT: Seated Medicine Ball Throw

Studies (year)	Design	Sport/players (n)	Protocol	Outcomes	Findings
Andhare et al. (2018) [19]	RCT	Cricket fast bowlers/40	Six weeks, 3×/week, standard T10 vs. control	Bowling performance and scapular function	Significant improvement in performance and shoulder function in T10 group
Franz et al. (2019) [20]	Feasibility/pilot	Youth baseball players/24	T10 taught to coaches; two-day training and six-week follow-up	Feasibility, coach accuracy, and implementation	Program feasible; coaches implemented with high accuracy (3×/week); supports injury-prevention applicability
Ponkshe et al. (2021) [21]	Pre-post experimental	Cricket bowlers/30	Six weeks, 3×/week, standard T10	LSST, FTPI, and throwing accuracy	Improved scapular control and throwing accuracy
Timothy and Subramanian (2023) [22]	Quasi-experimental	Cricket bowlers/82	Six weeks, 3×/week, T10 warm-up vs. standard warm-up	ROM, posterior capsule, internal rotator strength, CKCUEST, and SMBT	T10 warm-up group showed significant improvements in ROM, strength, and stability
Shah et al. (2024) [23]	Controlled trial	Handball players/60	Eight weeks, 3×/week, T10 vs. core vs. conventional	SMBT, FTPI, and plank test	T10 group showed superior improvements in SMBT, FTPI, and plank
Ramkumar et al. (2024) [24]	Experimental	Shot put athletes/15	Six weeks, T10+plyometric training	Grip strength, throwing distance	Combined T10+plyometric training improved grip strength and throwing distance
Tomar et al. (2024) [25]	Pre-post experimental	Badminton novice players/10	Six weeks, standard T10	SMBT, FTPI, and throwing accuracy	Significant improvements in throwing distance and accuracy
Bera et al. (2025) [26]	Experimental	Overhead athletes/30	Six weeks, advanced T10	Strength, power, and endurance	Significant improvements in upper-limb strength, power, and endurance

Study Characteristics

The included studies investigated 291 athletes across cricket, baseball, handball, badminton, shot put, and mixed overhead sports. Designs comprised RCTs, controlled trials, quasi-experimental studies, feasibility, and pre-post experimental designs. Intervention duration was typically six to eight weeks, performed three times per week.

Risk of bias assessments are summarized in Table 2. The randomized controlled trials demonstrated moderate-to-high methodological quality with PEDro scores ranging from 6 to 8 out of 10, indicating low risk of bias. Non-randomized and quasi-experimental studies scored between 6 and 7 on the Newcastle-Ottawa Scale, reflecting generally low-to-moderate risk of bias. Pre-post and feasibility studies evaluated with the JBI checklists achieved scores between 6 and 8 out of 9, suggesting acceptable methodological rigor.

**Table 2:** Risk of bias assessment of included studies. PEDro: Physiotherapy Evidence Database; JBI: Joanna Briggs Institute; NOS: Newcastle-Ottawa Scale

Studies	Design	Tool used	Score/rating	Risk of bias judgment
Andhare et al. (2018)	RCT	PEDro	7/10	Low risk
Franz et al. (2019)	Feasibility/pilot	JBI	7/9	Moderate risk
Ponkshe et al. (2021)	Pre-post study	JBI	8/9	Low risk
Timothy and Subramanian (2023)	Quasi-experimental	NOS	6/9	Moderate risk
Shah et al. (2024)	Controlled trial	NOS	7/9	Low risk
Ramkumar et al. (2024)	Experimental	NOS	6/9	Moderate risk
Tomar et al. (2024)	Pre-post study	JBI	7/9	Moderate risk
Bera et al. (2025)	Experimental	NOS	7/9	Low risk

Narrative Synthesis

Across studies, the Thrower’s Ten program and its adaptations were consistently associated with improved upper limb outcomes. In cricket bowlers, Andhare et al. and Ponkshe et al. reported enhanced scapular function, throwing accuracy, and performance [19,21]. Preventive benefits were also observed as follows: Franz et al. confirmed the feasibility and adoption of T10 in youth baseball [20], and Timothy and Subramanian demonstrated reduced posterior capsule tightness and improved ROM and stability when T10 was used as a warm-up [22].

Performance gains were documented in handball, badminton, shot put, and mixed overhead athletes. Shah et al. found T10 superior to core or conventional training for functional performance [23]. Ramkumar et al. showed that combining T10 with plyometric training enhanced grip strength and throwing distance in shot putters [24]. Tomar et al. observed improvements in throwing accuracy and functional tests in badminton players [25]. Finally, Bera et al. demonstrated that an advanced T10 protocol significantly increased strength, power, and endurance in overhead athletes [26].

Discussion

Overview of Findings

This systematic review synthesized evidence from eight studies published between 2015 and 2025 evaluating the Thrower’s Ten (T10) program in overhead athletes. The findings consistently support T10 as an effective intervention for improving shoulder function across cricket, handball, baseball, badminton, shot put, and mixed overhead sports. Across these diverse athletic populations, T10 was associated with improved scapular control, enhanced range of motion (ROM), reduced posterior capsule tightness, increased strength and endurance, and superior sport-specific performance. These findings reinforce the concept that shoulder health in overhead athletes depends not only on isolated rotator cuff strength but also on the integration of scapular stabilizers and the kinetic chain, as emphasized in contemporary shoulder rehabilitation models [1,3,14].

Rehabilitative Applications

The rehabilitative potential of the Thrower’s Ten program is strongly supported by evidence from cricket and badminton athletes. Andhare et al. demonstrated that cricket fast bowlers who performed six weeks of T10 showed significant improvements in scapular function and bowling performance compared with controls, emphasizing the program’s role in correcting scapular dyskinesis and restoring efficient kinetic chain mechanics [19]. Similarly, Ponkshe et al. reported that T10 training improved outcomes on the Lateral Scapular Slide Test (LSST) and Functional Throwing Performance Index (FTPI), both widely recognized measures of scapular alignment and functional overhead throwing performance [21]. Tomar et al. extended these findings to badminton players, noting significant gains in throwing accuracy and functional tests, suggesting that the benefits of T10 generalize beyond throwing-dominant sports into racquet sports where repetitive overhead strokes impose similar demands [25].

These improvements are clinically meaningful because scapular dyskinesis and glenohumeral internal rotation deficit (GIRD) are common precursors to injury in overhead athletes. GIRD, in particular, has been linked to increased risk of labral pathology and rotator cuff injury [13]. The T10 program directly addresses these deficits by targeting posterior capsule flexibility and internal rotation range of motion through integrated rotator cuff and scapular stabilizer strengthening. From a biomechanical perspective, overhead throwing exposes the shoulder to extreme external rotation in the late cocking phase and high eccentric demands on the posterior rotator cuff during deceleration [10-12]. Without adequate strength and endurance of these stabilizers, athletes are predisposed to impingement, labral tears, and instability. By simultaneously strengthening the rotator cuff and scapular stabilizers, while integrating multi-planar and kinetic chain elements, the T10 provides a more comprehensive rehabilitation strategy compared to traditional, isolated muscle strengthening [14,15].

Collectively, these findings reinforce T10 as a cornerstone rehabilitation tool. It not only restores deficits in scapular mechanics and posterior capsule flexibility but also improves functional performance relevant to return-to-play criteria, such as throwing accuracy and stability in closed kinetic chain tests. For clinicians, this evidence underscores that T10 can be used not only for shoulder pain management but also as a structured progression toward sport-specific rehabilitation goals.

Preventive Role

The preventive potential of the Thrower’s Ten program was demonstrated in both youth and adult athletes, highlighting its adaptability across age groups and training contexts. Franz et al. evaluated the feasibility of T10 implementation in youth baseball and found that coaches could accurately deliver the program, with players adhering to three sessions per week over six weeks [20]. This study is significant because it shows that T10 can be embedded into team settings without requiring specialist supervision, making it an accessible preventive strategy at the grassroots level. Timothy and Subramanian extended the preventive evidence to recreational cricket bowlers, showing that incorporating T10 into warm-ups resulted in reduced posterior capsule tightness, increased internal rotation range, and improvements in shoulder stability when compared to standard warm-ups [22]. These adaptations are critical because posterior capsule stiffness and reduced internal rotation are modifiable risk factors associated with overuse shoulder injuries in overhead athletes.

Taken together, these findings demonstrate that T10 can function as more than a rehabilitative tool; it can serve as a proactive, in-season, or preseason preventive strategy. The reductions in posterior capsule tightness and improvements in scapular control align with established prevention models proposed by Cools et al. and Kibler et al., which emphasize comprehensive kinetic chain conditioning to mitigate injury risk [1,14]. Importantly, T10 appears to combine the preventive benefits of structured warm-up routines with rehabilitative depth, offering a dual function not always achieved by other prevention programs, such as the “11+ shoulder” for handball. While the “11+” series focuses primarily on mobility and strength, T10 has broader applicability because it also serves rehabilitative purposes, allowing continuity of care from injury prevention through return to play.

In clinical and coaching practice, these findings suggest that integrating T10 into warm-up routines or preseason conditioning programs may reduce injury risk by addressing modifiable deficits before symptoms develop. Moreover, the program’s minimal equipment requirements make it highly feasible in team environments, schools, and youth.

Performance Enhancement

Although originally designed as a rehabilitation program, the Thrower’s Ten has demonstrated performance-enhancing benefits across several sports. Shah et al. found that handball athletes completing T10 achieved superior outcomes in functional performance tests, including plank endurance and the seated medicine ball throw, compared with core or conventional training [23]. Ramkumar et al. reported that combining T10 with plyometric training produced greater improvements in grip strength and throwing distance among shot putters, highlighting the value of integrated protocols for explosive performance [24]. Tomar et al. confirmed that even novice badminton players benefited from T10, showing better accuracy and functional performance measures [25]. Finally, Bera et al. demonstrated that an advanced variation of the program significantly enhanced strength, power, and endurance in mixed overhead athletes [26]. These findings suggest that T10, when progressed with higher resistance, plyometric elements, or advanced adaptations, can bridge rehabilitation and performance training by supporting both injury resilience and competitive capacity.

Strengths and Limitations of Evidence

A strength of the current body of evidence is its consistency; across all eight studies, T10 produced positive outcomes, regardless of sport or competitive level. The interventions were relatively short (six to eight weeks), required minimal equipment, and still resulted in measurable gains in function and performance. This highlights the practicality and accessibility of T10 in clinical and sporting environments. However, limitations must be acknowledged. Most studies were conducted with small samples, often fewer than 30 athletes, limiting statistical power. Methodological quality was variable, with only two randomized controlled trials scoring moderate to high quality on the PEDro scale, while quasi-experimental and pre-post studies often lacked allocation concealment and blinding. Protocol heterogeneity, such as differences between standard, advanced, and combined versions of T10, as well as variation in outcome measures, precluded meta-analysis. Furthermore, none of the studies included long-term follow-up, making it impossible to determine whether T10 reduces injury incidence or recurrence over competitive seasons.

Clinical Implications

The findings of this review have important implications for clinical practice. For physiotherapists, athletic trainers, and coaches, the Thrower’s Ten program offers a simple, low-cost, and adaptable intervention that can be applied across rehabilitation, prevention, and performance settings. It can be implemented to restore scapular alignment and rotator cuff balance during injury recovery, integrated into preseason warm-ups to reduce modifiable risk factors, and progressed into advanced or hybrid formats to enhance strength and power. A frequency of three sessions per week for six to eight weeks appears effective, but tailoring to baseline deficits and sport-specific demands is advisable. The minimal equipment requirement - elastic bands and light weights - makes T10 particularly feasible in youth programs and resource-limited environments, broadening its applicability beyond elite settings.

Future Research Directions

Future research should aim to address current gaps by conducting larger, multi-center randomized controlled trials that can confirm the effectiveness of T10 across sports. Longitudinal studies are particularly needed to evaluate the program’s impact on injury prevention, recurrence, and time lost from competition across an entire season. Studies exploring the minimal effective dose, sport-specific modifications, and applications in elite athletes with higher training loads would provide valuable insights. Economic analyses may also strengthen the case for widespread adoption by demonstrating cost-effectiveness relative to surgical or long-term rehabilitation pathways.

## Conclusions

This systematic review evaluated eight studies published between 2015 and 2025 that investigated the Thrower’s Ten program in overhead athletes. Evidence across cricket, handball, baseball, badminton, shot put, and mixed overhead sports shows that the program consistently improves scapular stability, shoulder range of motion, rotator cuff strength, endurance, and sport-specific performance. Originally developed as a rehabilitative protocol, the Thrower’s Ten has now demonstrated clear utility as a comprehensive intervention spanning rehabilitation, injury prevention, and performance enhancement. Its simplicity, accessibility, and adaptability make it a valuable evidence-based tool for clinicians, coaches, and athletes in overhead sports.
